# Cardiorespiratory Fitness Is an Indicator of Arterial Stiffness and Aortic Blood Pressure in Healthy Adolescents

**DOI:** 10.3390/children11091078

**Published:** 2024-09-02

**Authors:** Hwan Kim, Scott R. Collier, Valerio Bonavolontà, Austin Lassiter, Seaver Wait, Marco Meucci

**Affiliations:** 1 Department of Public Health and Exercise Science, Appalachian State University, Boone, NC 28608, USA; joj@appstate.edu (H.K.); colliersr@appstate.edu (S.R.C.); lassiterat@appstate.edu (A.L.); waitso@appstate.edu (S.W.); 2 Department of Biotechnological and Applied Clinical Sciences, University of L’Aquila, 67100 L’Aquila, Italy; valerio.bonavolonta@univaq.it

**Keywords:** pulse wave velocity, cardiorespiratory fitness, body mass index, fat mass, adolescents

## Abstract

**Background/Objectives:** We aimed to investigate the influence of cardiorespiratory fitness (CRF) and body composition on arterial stiffness. **Methods:** Carotid-to-femoral pulse wave velocity (cfPWV) and aortic systolic (ASBP) and diastolic (ADBP) blood pressure were compared between upper and lower tertiles of oxygen consumption at the aerobic threshold (VO_2AerT_), peak oxygen consumption (VO_2peak_), percentage of fat mass (FM%), and body mass index (BMI) in sixty adolescents (30 males and 30 females, 14.9 ± 2.1 years old). A stepwise multivariable linear regression analysis was performed to investigate the independent associations between VO_2AerT_ and VO_2peak_ and cfPWV, and between BMI and FM% and cfPWV with adjustments for age, sex, ASBP, and ADBP. **Results:** cfPWV and ADBP were lower in the second and third VO_2AerT_ tertiles compared to the first tertile (cfPWV, 4.7 ± 0.5 and 4.7 ± 0.5 vs. 5.3 ± 0.8 m/s, *p* < 0.01; ADBP, 62 ± 7 and 62 ± 7 vs. 70 ± 8 mmHg, *p* < 0.01). ASBP was lower in the third VO_2AerT_ tertile compared to the first tertile (94 ± 7 vs. 101 ± 12 mmHg, *p* = 0.05). ADBP was lower in the second VO_2peak_ tertile compared to the first tertile (62 ± 7 vs. 68 ± 9 mmHg, *p* = 0.03). ASBP was lower in the first and second BMI tertiles compared to the third tertile (95 ± 8 and 95 ± 7 vs. 102 ± 11 mmHg, *p* = 0.02). The eight-variable model significantly contributed to the variance of cfPWV (F(8, 51) = 7.450, *p* < 0.01), accounting for 47% of the variance. Individually, age (*p* < 0.05) and ADBP (*p* < 0.01) significantly predicted cfPWV. **Conclusions:** Submaximal indicators of CRF such as VO_2AerT_ should be considered as a part of the risk stratification of cardiovascular disease in healthy adolescents.

## 1. Introduction

The Center for Disease Control and Prevention (CDC) reports that cardiovascular disease (CVD) is the leading cause of death in the United States. Although the physical manifestation of this disease is less prevalent in adolescents, research shows that the development of CVD starts from childhood [1,2]. It is well known that the development of cardiovascular disease is multifactorial, and obesity, a sedentary lifestyle, and low cardiorespiratory fitness (CRF) are important risk factors for the development of CVD. In fact, risk factors such as obesity and physical inactivity in early life correlate with anatomical changes in the coronary arteries and aorta through the increase in atherosclerotic-related hypertension [3]. Research shows that overweight and obese children with high percentages of fat mass (FM%) and a body mass index (BMI) above the 85^th^ percentile also report higher arterial stiffness and blood pressure compared to their lean peers, placing them at a higher risk of developing hypertension and CVD later in life [4,5]. Specifically, excess adipose tissue has been associated with systemic inflammation [6], which influences the progression of atherosclerosis leading to CVD [7]. Yet, regular exercise can increase CRF and delay obesity-related comorbidities by modulating the inflammatory processes at all ages. In fact, CRF in children is negatively associated with low-grade inflammatory markers, which is positively associated with body fat [8].

Non-invasive assessments of aortic blood pressure have shown to predict cardiovascular events better than brachial assessments [9]. Carotid-to-femoral pulse wave velocity (cfPWV) is considered the gold standard for the assessment of central arterial stiffness in children [10], and it is a valid biomarker for the diagnoses and risk stratification of CVD [11]. Another key CVD risk factor in children is low CRF. Although measurements of maximal aerobic capacity (expressed as peak oxygen consumption (VO_2peak_)) are considered the best indicator of CRF in pediatric populations, submaximal measurements of aerobic efficiency such as the oxygen consumption at the aerobic threshold (AerT) are considered an adequate biomarker of fitness in youth [12]. Submaximal indicators of CRF are commonly used in children since young individuals may lack motivation to exercise at severe intensities and/or fail to deliver maximal effort [12]. Research shows that values of oxygen consumption at AerT lower than 11 ml/kg/min are associated with an increased risk of death in the clinical population [13]. Moreover, CRF is inversely associated with arterial stiffness, independently of fat mass [14], and high levels of CRF have been shown to decrease the incidence of all-cause cardiovascular disease [15]. Furthermore, it has been suggested that sufficiently high CRF may attenuate the deleterious cardiovascular effects of obesity in adults, providing a stepping stone for the “fit but fat” paradigm [16]. However, evidence in support of such a paradigm, or observations made in children and adolescents, is inconsistent, in part due to limited access to obese yet healthy adolescents.

Therefore, this study aimed to investigate the association between non-invasive, gold-standard indicators of CRF and the body fat percentage with cfPWV and the central arterial pressure in healthy adolescents. 

## 2. Materials and Methods

### 2.1. Participants

Sixty adolescent to middle-adolescent children (30 males and 30 females) aged 11 to 17 years were recruited for this study. To ensure socioeconomic and ethnic diversity in the research sample, participants were recruited from a no-cost, state-funded program for academically talented students drawn from various rural and urban communities across North Carolina, USA and from the local community. Recruitment efforts included the distribution of flyers and word of mouth. Both the adolescents and their parents/guardians provided written assent and consent to participate in the research. To ensure the health and safety of the participants, exclusion criteria were applied, including the presence of medical conditions such as diabetes or cardiovascular, respiratory, or renal diseases and the use of any medications at the onset of this study. Participants (and their parents or guardians) were advised to report to the laboratory at least 3 h post consumption of caffeinated beverages and large meals and to refrain from exercise the day prior to the scheduled testing sessions. Measurements were conducted during two different time windows, either 8:00 to 11:00 AM or 4:00 to 6:00 PM, to accommodate various schedules and ensure consistency in data collection.

### 2.2. Anthropometrics and Body Composition

Height and body mass were precisely measured using a stadiometer and a calibrated scale to the nearest 0.1 cm and 0.1 kg, respectively. Body mass index (BMI) was subsequently calculated using the standard formula: BMI = weight (kg)/height^2^ (m^2^). Body composition, including fat mass (FM) and fat-free mass (FFM), was assessed for all participants using air displacement plethysmography (BodPod Gold, COSMED, Rome, Italy). The Bod Pod reports acceptable (1–2% difference from the gold standard) [17] and reliable FM% measurements [18] and it provides a fast, non-operator-dependent, and minimally invasive body composition assessment. Both absolute values and percentages relative to total body weight (FM% and FFM%) were derived. The assessments were conducted by employing the Siri equation and using the predicted thoracic gas volume. During the body composition evaluation, subjects were required to wear tight-fitting clothing and a swimmer’s cap and to remove shoes and any jewelry, to ensure accurate and consistent results.

### 2.3. Cardiovascular Measurement

Blood pressure and arterial stiffness were evaluated using the automated SphygmoCor XCEL system (SphygmoCor, AtCor Medical, Inc. Naperville, IL, USA. Brachial blood pressure measurements were taken from the right arm after 5 min of quiet rest, with the participants lying supine in a dimly lit room to ensure a relaxed state. cfPWV was calculated as the ratio of the vascular distance to the pulse wave transit time, expressed in meters per second (m/s). The SphygmoCor XCEL software version 1.3 determined the vascular distance based on the measurements from the carotid artery to the sternal notch, the sternal notch to the leg cuff, and the femoral artery to the leg cuff. Pulse wave transit time was measured using volumetric displacement for the femoral artery and applanation tonometry for the carotid artery. These distances were measured with a fabric tape measure by a trained technician. To ensure accuracy, recordings of the waveforms with consistent amplitudes and shapes that met or exceeded a quality control index of 90% were included in the statistical analysis. Each participant underwent a minimum of three measurements, with one-minute rest intervals between each successive measure. The final data point was given as the average of the two closest trials, provided they met the following criteria: systolic and diastolic blood pressure within ±5 mmHg for PWA [19] and cfPWV measurements within ±0.3 m/s for PWV [20]. 

### 2.4. Cardiorespiratory Fitness Test

The CRF was evaluated using an incremental exercise test on an electronically braked pediatric cycle ergometer (Lode Corrival, Groningen, Netherlands). The cycle ergometer was selected in this study because it allows precise control of exercise intensity while minimizing the risk of injury and improving ease of exercise especially for overweight/obese participants. The test protocol commenced with a 2-min resting period while seated on the ergometer, followed by 1 min of unloaded pedaling at 0 W. Thereafter, the exercise intensity increased by 15 W per minute, maintaining a cadence of 65–70 revolutions per minute (rpm), until the participant could no longer sustain a minimum cadence of 50 rpm or chose to stop. Respiratory gas exchange was continuously monitored breath-by-breath using a calibrated metabolic cart (K5, Cosmed, Chicago, IL, USA). Before each test, turbine calibration (using a 3-liter syringe), gas calibration (using 16.00% and 20.93% O_2_ and 5.0% and 0.04% CO_2_ gas concentrations), CO_2_ scrubber calibration, and delay calibration were performed as per the manufacturer’s guidelines [21]. The raw breath-by-breath data were processed using a 6-point moving average smoothing technique, and then averaged over 10-s intervals for all variables. Heart rate (HR) was continuously recorded throughout the test using a GARMIN chest strap heart rate monitor (GARMIN, Olathe, KS, USA).

### 2.5. Aerobic and Anaerobic Thresholds

VO_2_ (ml/kg/min) at AerT (VO_2AerT_) and AnT (VO_2AnT_) was determined graphically using a combination of the ventilatory equivalent method and the V-slope method as described by Mayer et al. (2005) [22]. The ventilatory equivalent method was used as the primary criterion, with the V-slope method serving as a secondary criterion for cross-validation. The AerT and AnT were determined independently by the two principal investigators and, if results varied more than 30 s, the opinion of an experienced exercise physiologist was used to agree on a result. VO_2AerT_ was computed as the 30-s average of VO₂ around this agreed time point and expressed in ml/kg/min. VO_2peak_ was determined as the 30-s average of the highest VO₂ values recorded during the final minute of the incremental exercise test. The original data can be found in the Supplementary Materials.

### 2.6. Statistical Analysis

Differences in descriptive characteristics and in cardiovascular measurements between male and female participants were investigated using a one-way analysis of variance (ANOVA). The independent associations between indices of CRF (VO_2AerT_ and VO_2peak_), adiposity parameters (BMI and FM%), and cfPWV were assessed using a stepwise multivariable linear regression analysis with adjustments for age, sex, ASBP, and ADBP. Sex did not attenuate any of the investigated associations (adjusted *B* = 0.038, t = 0.277, *p* = 0.783). Therefore, we performed further analyses with male and female participants combined. To analyze the relationship of different fitness and body composition metrics with cardiovascular outcomes, our sample of adolescents was divided into tertiles based on VO_2AerT_, VO_2peak_, BMI, and FM%. With an a priori power analysis for comparing two independent means of cfPWV using data previously published from our laboratory, a two-tailed test, an anticipated effect size of 0.907, an alpha level of 0.05, and a desired power of 0.8, we determined that a sample size of 21 participants per group was necessary to detect a statistically significant difference. Each metric was stratified into the lowest (first tertile), middle (second tertile), and highest (third tertile) thirds. Differences in cfPWV, aortic systolic blood pressure (ASBP), and aortic diastolic blood pressure (ADBP) between tertiles of BMI, FM%, VO_2AerT_, and VO_2peak_ were assessed using a one-way ANOVA with Sidak post hoc tests for multiple comparisons of tertiles for each dependent variable, with each comparison evaluated at an alpha level of 0.05. To further explore the relationship between body composition and cfPWV, participants were classified into three groups based on their FM%. These groups included 1) Mid FM%: Participants with FM% within one standard deviation (SD) from the cohort mean (*n* = 38), 2) Low FM%: Participants with FM% below one standard deviation from the mean (*n* = 12), and 3) High FM%: Participants with FM% above one standard deviation from the mean (*n* = 10). The differences in cfPWV among these three FM% groups were assessed via ANOVA. The alpha level was set at 0.05 for statistical significance. All analyses were performed using SPSS Software version 29.0.1.0(171), IBM. The level of significance was set at *p* < 0.05. Results are expressed as the mean ± SD.

## 3. Results

**Figure 1:**
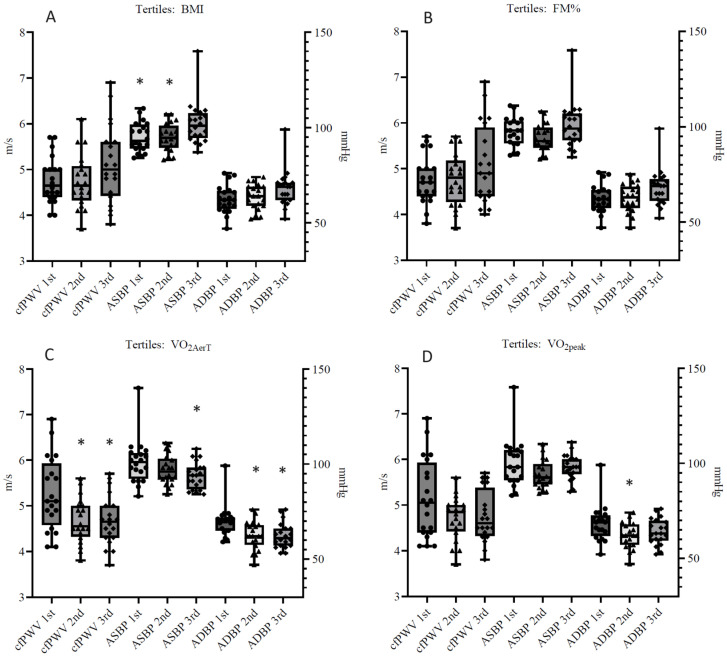
Differences in cardiovascular measures between BMI, FM%, VO_2max_ and VO_2AerT_ tertiles. (**A**,**B**), * *p* < 0.05 compared to the third tertile. (**C**,**D**), * *p* < 0.05 compared to the first tertile.

Participants’ descriptive characteristics are reported in Table 1. The Kolmogorov–Smirnov test for normality conducted on cfPWV data from 60 participants did not reject the null hypothesis of normality (*p* = 0.09), suggesting that the data were consistent with a normal distribution. The sample for this study was comprised of individuals representing a diverse range of ethnicities including white (*n* = 35, 58.3%), Indian (*n* = 12, 20.0%), Asian (*n* = 9, 15.0%), and Black (*n* =4, 6.7%). Eighteen subjects were considered overweight or obese (8 overweight and 10 obese) as per US Centers for Disease Control and Prevention BMI-for-age growth charts (58.5 ± 30.9 BMI percentile). Males in this sample had a higher height (*p* < 0.01, F = 13.7), body mass (*p* < 0.01, F = 8.3), FFM% (*p* < 0.01, F = 21.2), ASBP (*p* = 0.01, F = 7.1), and VO_2peak_ (*p* = 0.03, F = 4.8) than the females. Females had a higher HR at AerT (HR_AerT_) (*p* < 0.01, F = 11.1) and HR at AnT (HR_AnT_) (*p* < 0.01, F = 7.3) compared to males. Differences in cardiovascular measures between VO_2AerT_, VO_2peak_, BMI, and BF% tertiles are reported in Table 2, Table 3, Table 4 and Table 5 and in Figure 1, respectively.

**Figure 2:**
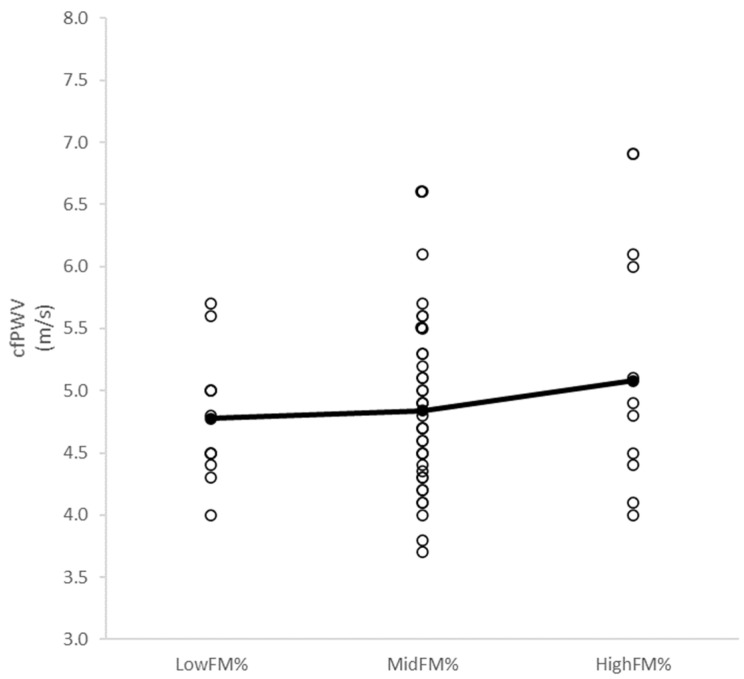
Differences in cfPWV between LowFM%, MidFM% and HighFM% groups.

ASBP was higher in the third BMI tertile compared to the first two (*p* = 0.02, F = 4.4), and no differences were found between BMI and FM% tertiles in any cardiovascular measure. The second and third VO_2AerT_ tertiles had lower cfPWV (*p* < 0.01, F = 5.6) and ADBP (*p* < 0.01, F = 6.5) compared to the first tertile. The third VO_2AerT_ tertile had lower ASBP (*p* = 0.05, F = 3.1) compared to the first tertile. The second VO_2peak_ tertile had lower ADBP (*p* = 0.03, F = 3.6) compared to the first tertile. No differences in cfPWV (*p* = 0.53, F = 0.6) were observed between LowFM%, MidFM%, and HighFM% (4.8 ± 0.5 m/s, 4.8 ± 0.6 m/s, and 5.1 ± 1.0 m/s, respectively) (Figure 2).

Results of the multivariable analysis are reported in Table 6. The analysis showed that the eight-variable model significantly contributed to the variance of cfPWV (F(8, 51) = 7.450, *p* < 0.01), accounting for 47% of the variance. Individually, two out of the eight variables significantly predicted cfPWV. Participants more likely had a higher cfPWV with a higher ADBP (adjusted *B* = 0.389, t = 2.321, *p* < 0.01) and if they were older (adjusted *B* = 0.420, t = 3.053, *p* < 0.05). VO_2AerT_, VO_2peak_, BMI, and BF% were not independently associated with cfPWV.

## 4. Discussion

In our cohort of adolescents, we observed that age and ADBP were the best predictors of higher arterial stiffness independently of sex and that children with higher levels of CRF had lower cfPWV and reduced central blood pressure. Interestingly, our findings indicated that elevated FM% did not uniformly correspond to increased cfPWV, and there was a tendency toward an association between BMI and cfPWV, with the correlation approaching statistical significance. This suggests that CRF might serve as a more robust indicator of cardiovascular health in healthy adolescents compared to BMI and FM%. Also, our data suggest that VO_2AerT_ could be a better indicator of cardiovascular health compared to VO_2peak_ in apparently healthy, recreationally active adolescents. Therefore, we postulate that VO_2AerT_, an index of aerobic efficiency, may play an important role in the association between overweight and cardiovascular health in adolescents.

Previous research has shown the association between high CRF and lower central arterial stiffness and systolic blood pressure in children [14,23], and that overweight and obese children tend to display high levels of arterial stiffness and elevated blood pressure [24]. These findings align with our results, which indicate that adolescents with higher CRF have lower cfPWV and aortic blood pressure. In contrast, adolescents with a high BMI exhibit elevated ASBP, but not cfPWV, compared to their leaner peers. Surprisingly, aortic blood pressure and cfPWV did not differ among adolescents with varying FM%. Although obesity is an independent predictor of cardiovascular risk, the direct association between excess adiposity and arterial stiffness has previously been debated, especially in healthy children and adolescents [25]. Previous studies have shown that obese children often suffer from elevated blood pressure and hypertension, which may alter arterial functions and increase arterial stiffness [26]. Although increased adiposity is linked to attenuated adiponectin signaling [27] and increased inflammatory cytokines concerning endothelial dysfunction and arterial stiffness in children [28,29], this process does not always manifest phenotypically, especially in adolescents with high levels of CRF. In fact, overweight and obese adolescents can show low arterial stiffness due to the cardioprotective effect of exercise, since CRF is inversely and independently associated with arterial stiffness [14,30]. Research conducted in adult populations shows CRF may be a stronger modulator of cardiovascular risk than body composition [31], yet the benefits of high fitness levels may be compromised by obesity. Although this finding is promising, obese children may respond to higher fitness levels differently than adults. Recently, a systematic review confirmed the inverse association between CRF and central PWV. Also, there were inconsistencies between adolescents and adults since a positive association between fitness and central PWV in adolescents was only observed in the adolescents [32]. Additionally, a study conducted on 252 male children and adolescents reported that body weight determines central systolic blood pressure, a surrogate for arterial stiffness, rather than CRF [33]. The divergent findings could be caused by the method used to determine CRF (estimations vs. direct measurements) and/or too wide an age range, including children in different developmental stages, and/or the inclusion of participants with a relatively low body weight and high level of CRF. Therefore, it is essential to include participants with a wide range of body weights and CRFs, and to use direct assessment of CRF in the evaluation of cardiovascular risk factors in healthy, young individuals [34]. VO_2peak_ is considered the gold standard for CRF. However, it may not be the best way to assess the aerobic capacity in children as psychological and physiological factors can limit the achievement of VO_2peak_ in this population. Research shows that a lack of motivation to reach the maximal effort [12,35] and an undeveloped anaerobic metabolism [36] may increase the margin of error while assessing VO_2peak_, thus calling into question its utility in children and adolescents for preventing the achievement of maximal effort. Moreover, the achievement of VO_2peak_ and VO_2AerT_ have shown to be independent in children and do not occur at the same rate. While VO_2peak_ mainly relies on both a maximal cardiac output and oxygen extraction, VO_2AerT_ depends on the muscle oxidative capacity [12] and the ability to produce and remove lactate, which is impaired in young children [37,38]. This indicates that VO_2AerT_ may be more appropriate than VO_2peak_ for assessing CRF, and it can be identified even if individuals prematurely end the graded exercise test. When considering strategies to improve CRF, research shows that moderate exercise intensities can provide enough of a stimulus to improve both indices of fitness and health [39]. However, voluntary physical activity may lack the duration and intensity associated with adequate stimuli for chronic aerobic exercise adaptations in children [40]. In contrast, research conducted by our laboratory shows that when play-based physical activity is supervised, improvements in CRF and cardiovascular health in overweight children can occur, independently of changes in body weight and body composition [41]. 

Another important finding of this study is that cardiovascular measures showed a decreasing trend across both VO_2peak_ and VO_2AerT_ tertiles. However, mean differences were significant only in VO_2AerT_ tertiles. This result suggests that VO_2peak_ may not be the best indicator of CRF in adolescents, as young individuals may reach volitional exhaustion before the maximal oxygen consumption is attained [42]. On the contrary, submaximal indicators of CRF such as VO_2AerT_ may be preferred, as they have been shown to be effective indicators of cardiovascular health [11]. This result is highly relevant in the pediatric field as the prognosis of cardiovascular disease in adolescents can be acquired using submaximal indicators of CRF. Moreover, it is possible to increase VO_2AerT_ in children with just a moderate exercise intensity [43], while a vigorous exercise intensity is necessary to improve VO_2max_ [44]. It may be more important to consider clinical relevance between tertiles even if mean differences are not statistically significant. For example, children and adolescents are considered hypertensive when their blood pressure readings are ≥95^th^ percentile for sex, age, and height with 4 mmHg difference between the 90^th^ and 95^th^ percentiles in both systolic and diastolic blood pressure [45]. Although not statistically significant, the first and second tertiles of BMI and FM% showed a mean difference in aortic pressures equal or greater than 4 mmHg against the third tertile. Similarly, the second and third tertiles of VO_2peak_ and VO_2AerT_, when not statistically different, showed similar mean differences in aortic blood pressure when compared to the first tertile. These results are relevant as they suggest that adolescent children with VO_2AerT_ and VO_2peak_ above 14.5 ml/kg/min and 30 ml/kg/min, and BMI and FM% below 23kg/m^2^ and 30%, respectively, may be at a lower risk of developing high blood pressure and consequently arterial stiffness.

This research study included participants from different ethnic backgrounds, with the ethnic distribution closely mirroring the demographic composition of North Carolina and of the general United States population (≈60% white, 6–20% Indian, Asian, and African American). Although the percentage distributions of the sample did not perfectly match those of this state and country, it is important to consider how the sample’s diversity supports the generalizability of the results. We recognize the limitations of our methods, including the heterogeneous sample of males and females, the limited number of overweight/obese participants, and the potential selection bias. Although our sample had an equal number of male and female participants, the two sexes were not equally distributed between tertiles and we recognize that sex differences were not analyzed in this study. Moreover, only one-third of our sample (18 out of 60 participants) was overweight/obese. A larger representation of overweight and especially obese individuals may help in investigating differences in cardiovascular measures in individuals with high BMI and FM%. Finally, this study’s potential selection bias, due to the exclusion of participants with certain medical conditions, may have skewed the results by not fully representing the diverse range of health profiles, thereby limiting the generalizability and applicability of the findings. Our findings are limited to cross-sectional data and do not provide further evidence of the mechanisms by which CRF may attenuate the deleterious cardiovascular effects of overweight or excess adiposity beyond the healthy normative values in adolescents. Future studies should address these limitations by recruiting more overweight and obese individuals and monitoring CRF before and after specific physical exercise programs in this population. Specifically, investigating the effect of different types of physical activity on CRF and their impact on CV health may help us to further understand the dose–response relationship between aerobic fitness and arterial health in overweight adolescents.

## 5. Conclusions

In conclusion, VO_2AerT_ may be an appropriate indicator of cardiovascular risk in healthy adolescents. Future research should validate and extend the utility of CPET maximal versus non-maximal efforts in clinical pediatric and adult populations. Moreover, future interventions using light-to-moderate intensity physical activity, such as play-based activity, to improve the VO_2AerT_ should be conducted to investigate how improvements in cardiorespiratory efficiency can improve cardiovascular outcomes in this population. 

## Figures and Tables

**Table 1:** Descriptive characteristics and cardiovascular measures of adolescent males and females.

Characteristics	All (*n* = 60)	Males (*n* = 30)	Females (*n* = 30)
Age (years)	14.9 ± 2.1	14.8 ± 2.2	15.0 ± 2.1
Height (cm)	165.5 ± 11.3	170.4 ± 12.0	160 ± 8.1 **
Body mass (BM; kg)	62.2 ± 19.8	69.1 ± 24.0	55.2 ± 10.9 **
BMI (kg/m^2^)	22.4 ± 5.4	23.5 ± 6.7	21.2 ± 3.6
FM% (%)	24.6 ± 11.4	22.5 ± 13.6	26.7 ± 8.4
FFM% (%)	75.4 ± 11.4	77.5 ± 13.6	73.3 ± 8.4 **
cfPWV (m/s)	4.9 ± 0.7	4.9 ± 0.7	4.8 ± 0.6
ASBP (mmHg)	97.2 ± 9.4	100 ± 10	94.2 ± 7.7 **
ADBP (mmHg)	64.6 ± 8.2	65 ± 9	64.4 ± 7.3
VO_2AerT_ (ml/kg/min)	17.2 ± 4.4	17.1 ± 3.9	17.3 ± 4.9
HR_AerT_ (bpm)	122 ± 16	116 ± 11	129 ± 18 **
VO_2AnT_ (ml/kg/min)	26.4 ± 6.5	27.0 ± 6.8	25.8 ± 6.1
HR_AnT_ (bpm)	158 ± 17	152 ± 17	163 ± 16 **
VO_2peak_ (ml/kg/min)	33.6 ± 7.9	35.8 ± 8.7	31.4 ± 6.5 *
HR_peak_ (bpm)	187 ± 12	188 ± 13	186 ± 12
Test duration (min)	11.5 ± 3.0	13.1 ± 3.0	9.9 ± 2.1 **

BMI, body mass index; FM%, fat mass percentage; FFM%, fat-free mass percentage; cfPWV, carotid femoral pulse wave velocity; ASBP, aortic systolic blood pressure; ADBP, aortic diastolic blood pressure; VO_2AerT_, VO_2_ at the aerobic threshold; HR_AerT_, heart rate at the aerobic threshold; VO_2AnT_, VO_2_ at the anaerobic threshold; HR_AnT_, heart rate at the anaerobic threshold; VO_2peak_, VO_2_ at peak; HR_peak_, heart rate at peak. Values are reported as the mean ± SD. * *p* < 0.05 compared to males. ** *p* < 0.01 compared to males.

**Table 2:** Differences in cardiovascular measures between VO_2AerT_ tertiles.

	First (*n* = 20)	Second (*n* = 20)	Third (*n* = 20)
cfPWV (m/s)	5.3 ± 0.8	4.7 ± 0.5 *	4.7 ± 0.5 *
ASBP (mmHg)	101 ± 12	97 ± 8	94 ± 7 *
ADBP (mmHg)	70 ± 8	62 ± 7 *	62 ± 7 *

First tertile, lowest VO_2AerT_; third tertile, highest VO_2AerT_. cfPWV, carotid-to-femoral pulse wave velocity; ASBP, aortic systolic blood pressure; ADBP, aortic diastolic blood pressure. * *p* < 0.05 compared to the first tertile.

**Table 3:** Differences in cardiovascular measures between VO_2peak_ tertiles.

	First (*n* = 20)	Second (*n* = 20)	Third (*n* = 20)
cfPWV (m/s)	5.1 ± 0.9	4.7 ± 0.5	4.8 ± 0.6
ASBP (mmHg)	100 ± 13	94 ± 7	98 ± 7
ADBP (mmHg)	68 ± 9	62 ± 7 *	64 ± 7

First tertile, lowest VO_2peak_; third tertile, highest VO_2peak_. cfPWV, carotid-to-femoral pulse wave velocity; ASBP, aortic systolic blood pressure; ADBP, aortic diastolic blood pressure. * *p* < 0.05 compared to the first tertile.

**Table 4:** Differences in cardiovascular measures between BMI tertiles.

	First (*n* = 20)	Second (*n* = 20)	Third (*n* = 20)
cfPWV (m/s)	4.7 ± 0.5	4.8 ± 0.6	5.1 ± 0.9
ASBP (mmHg)	95 ± 8 *	95 ± 7 *	102 ± 11
ADBP (mmHg)	62 ± 7	64 ± 7	68 ± 10

First tertile, lowest BMI; third tertile, highest BMI. cfPWV, carotid-to-femoral pulse wave velocity; ASBP, aortic systolic blood pressure; ADBP, aortic diastolic blood pressure. * *p* < 0.05 compared to the third tertile.

**Table 5:** Differences in cardiovascular measures between FM% tertiles.

	First (*n* = 20)	Second (*n* = 20)	Third (*n* = 20)
cfPWV (m/s)	4.8 ± 0.5	4.8 ± 0.6	5.1 ± 0.9
ASBP (mmHg)	97 ± 7	94 ± 7	101 ± 12
ADBP (mmHg)	63 ± 7	63 ± 7	68 ± 10

First tertile, lowest FM%; third tertile, highest FM%. cfPWV, carotid-to-femoral pulse wave velocity; ASBP, aortic systolic blood pressure; ADBP, aortic diastolic blood pressure.

**Table 6:** Association between adiposity/CRF and cfPWV in multivariable models.

Variables	Covariates	*B*	Adjusted R^2^	t	*p*	95% CI
FM%	Age, sex	−0.224	0.050	−1.029	0.309	−0.039 to 0.013
BMI	Age, sex	0.448	0.200	1.775	0.082	−0.007 to 0.119
VO_2peak_	Age, sex	−0.305	0.093	−1.451	0.153	−0.001 to 0.001
VO_2aerT_	Age, sex	0.014	0.000	0.092	0.927	−0.001 to 0.000

BMI, body mass index; FM%, fat mass percentage; VO_2AerT_, VO_2_ at the aerobic threshold; VO_2peak_, VO_2_ at peak.

## Data Availability

The original contributions presented in the study are included in the Supplementary Materials, further inquiries can be directed to the corresponding author/s.

## Supplementary Materials

The following supporting information can be downloaded at: https://www.mdpi.com/article/10.3390/children11091078/s1, Table S1: Body measurement data in sixty adolescents.
